# DFT‐Guided Synthesis, Electrochemical, and Photophysical Properties of Ruthenium(II) Polypyridyl Complexes Featuring Flavin‐Inspired π‐Extended Ligands

**DOI:** 10.1002/chem.202404627

**Published:** 2025-06-01

**Authors:** Nina Hagmeyer, Nabil Mroweh, Alexander Schwab, Caitilin McManus, Maneesha Varghese, Jean‐Marie Mouesca, Serge Gambarelli, Stephan Kupfer, Benjamin Dietzek‐Ivanšić, Murielle Chavarot‐Kerlidou

**Affiliations:** ^1^ Institute of Physical Chemistry and Abbe Center of Photonics Friedrich Schiller University Jena Helmholtzweg 4 Jena 07743 Germany; ^2^ Department Functional Interfaces Leibniz Institute of Photonic Technology Jena (IPHT) Albert‐Einstein‐Straße 9 Jena 07745 Germany; ^3^ Leibniz Institute of Surface Engineering (IOM) Permoserstraße 15 Leipzig 04318 Germany; ^4^ Center for Energy and Environmental Chemistry Jena (CEEC Jena) Friedrich Schiller University Jena Philosophenweg 8 Jena 07743 Germany; ^5^ Univ. Grenoble Alpes, CNRS, CEA, IRIG, Laboratoire de Chimie et Biologie des Métaux Grenoble 38000 France; ^6^ Univ. Grenoble Alpes, CNRS, CEA, IRIG, SyMMES Grenoble 38000 France

**Keywords:** alloxazine, artificial photosynthesis, flavin, ruthenium polypyridyl complex, π‐extended ligand

## Abstract

Light‐driven electron transfer and subsequent multielectron storage is among the key aspects of photochemical reactions in artificial photosynthesis and molecular electronics. Following our previously introduced design and characterization of Ru(II)‐based photosensitizers, four new Ru complexes with π‐extended ligands featuring a flavin‐inspired subunit were investigated via density functional theory in order to evaluate their electrochemical properties ahead of a time and resource‐demanding synthesis. Two complexes, **Ru‐Me_2_allox^B^** and **Ru‐Me_2_deazaallox^B^**, with a bent ligand architecture, were identified as promising candidates for application in light‐driven charge accumulation and subsequently synthesized. The electrochemical characterization of **Ru‐Me_2_allox^B^** confirmed the theoretical predictions and its photophysical properties were investigated using UV/Vis absorption, resonance Raman, time‐resolved emission, and time‐resolved absorption spectroscopy in combination with quantum chemical simulations. Furthermore, first insights into the electronic distribution in the singly reduced complex were modelled computationally and obtained by EPR and UV/Vis absorption spectroscopy and spectroelectrochemistry. These results underline the promising multielectron storage capacity of the newly designed π‐extended alloxazine ligand.

## Graphical abstract

The photophysical properties as well as the multielectron storage capacity of a several ruthenium(II) polypyridyl complexes with flavin‐inspired π‐extended ligands are investigated in a joint synthetic‐spectroscopic‐theoretical approach.

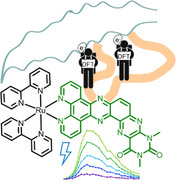

## 1 Introduction

Natural flavins are ubiquitous biological cofactors, involved in a wide range of photobiological processes, as well as single and multielectron transfer reactions thanks to their isoalloxazine structural unit, which can exist as three different redox‐active states. Such rich reactivity has motivated the design and study of bioinspired synthetic compounds^[^ ^1^ ^]^ either based on the isoalloxazine motif or on its alloxazine tautomer for energy‐related applications ranging from photocatalysis^[^ ^2^ ^]^ to redox‐flow batteries.^[^ ^3^, ^4^ ^]^ Modulation of the redox and photophysical properties of flavin compounds is therefore of great interest to fine‐tune their reactivity and broaden the scope of their applications. Synthetic modifications being quite challenging for this class of molecules, structural diversity has mainly focused on the nature of the alkyl substituents and lateral chains present on the (iso)alloxazine ring.^[^ ^2^, ^5^, ^6^, ^7^, ^8^ ^]^ Only a few more sophisticated strategies based on the extension of the aromatic π system and/or its coordination to a metal center have been described to date.^[^ ^9^, ^10^, ^11^, ^12^ ^]^ Annulation of benzene, naphthalene, or pyrene units to the parent flavin core was for instance reported by the groups of Webster and König to significantly affect the electrochemical reactivity, as well as the photophysical properties.^[^ ^10^ ^]^ An example is the bathochromic shift of the chromophore absorption due to the extended aromatic system.^[^ ^10^ ^]^ In a different approach, the ability of flavin and alloxazine compounds to act as redox‐active ligands to coordinate various transition metal centers through their O^4^─N^5^ sites has been extensively investigated in the 90s.^[^ ^13^ ^]^ Appending an additional coordination site to the (iso)alloxazine ring is another relevant but less explored strategy to develop (iso)alloxazine‐based ligands. This can be, for example, pyridine^[^ ^14^ ^]^, or catechol^[^ ^15^ ^]^ subunits but also bipyridine (bpy) or phenanthroline ones, which open the way to the preparation of photoactive flavin‐based Ru polypyridyl complexes. The group of Dick connected a bpy ligand to a flavin moiety via an acetylene‐based linker and investigated the photophysical properties of the corresponding Ru(II) complex.^[^ ^16^, ^17^ ^]^ The absorption in the visible region was enhanced but their complex displayed a slightly shorter excited‐state lifetime and a lower emission quantum yield compared to the [Ru(bpy)_3_]^2+^ prototype. Notably, they identified the lowest‐lying triplet state as ^3^IL instead of a ^3^MLCT conventionally observed for Ru polypyridyl complexes such as [Ru(bpy)_3_]^2+^ and [Ru(bpy)_2_(dppz)]^2+^. This highlights the need to design new assemblies to take better advantage of the electronic interplay of the two chromophores.

On the other hand, the electrochemical and spectroscopic properties of a Ru(II) tris‐diimine complex featuring an alloxazine‐based ligand, built by π‐extension of a phenanthroline with a pteridinedione unit, were reported by the group of McGuire;^[^ ^18^ ^]^ its photophysical properties as well as light‐driven reactivity were very recently investigated by our consortium,^[^ ^11^ ^]^ showing that the multielectron redox‐activity of the alloxazine subunit is key for light‐driven charge storage in the structure. We also previously designed a series of Ru(II) polypyridyl complexes with π‐extended dppz ligands featuring a bio‐inspired quinone‐like subunit,^[^ ^19^, ^20^, ^21^, ^22^ ^]^ highlighting that extended π‐conjugation can be a strategy of choice to develop original photoactive systems for energy conversion and storage applications.

In the study at hand, a similar approach was used to append an alloxazine subunit to the photoactive ruthenium center. A predictive approach relying on calculated redox potentials successfully guided the synthesis of two novel structures. The spectroscopic and photophysical properties of the complex displaying the most promising electrochemical properties were thoroughly investigated with the help of (time dependent) density functional theory ((TD)DFT) calculations and UV/Vis and resonance Raman (rR) spectroscopy. The excited‐state dynamics of the complex were elucidated via time‐resolved emission and transient absorption (TA) spectroscopy. Additionally, the electronic distribution in the singly reduced species was investigated by electronic paramagnetic resonance (EPR) spectroscopy, UV/Vis absorption spectroscopy and spectroelectrochemistry as well as (TD)DFT.

## 2 Results and Discussion

### 2.1 Molecular Design and DFT‐Guided Selection of the Most Promising Complexes

**Figure 1:**
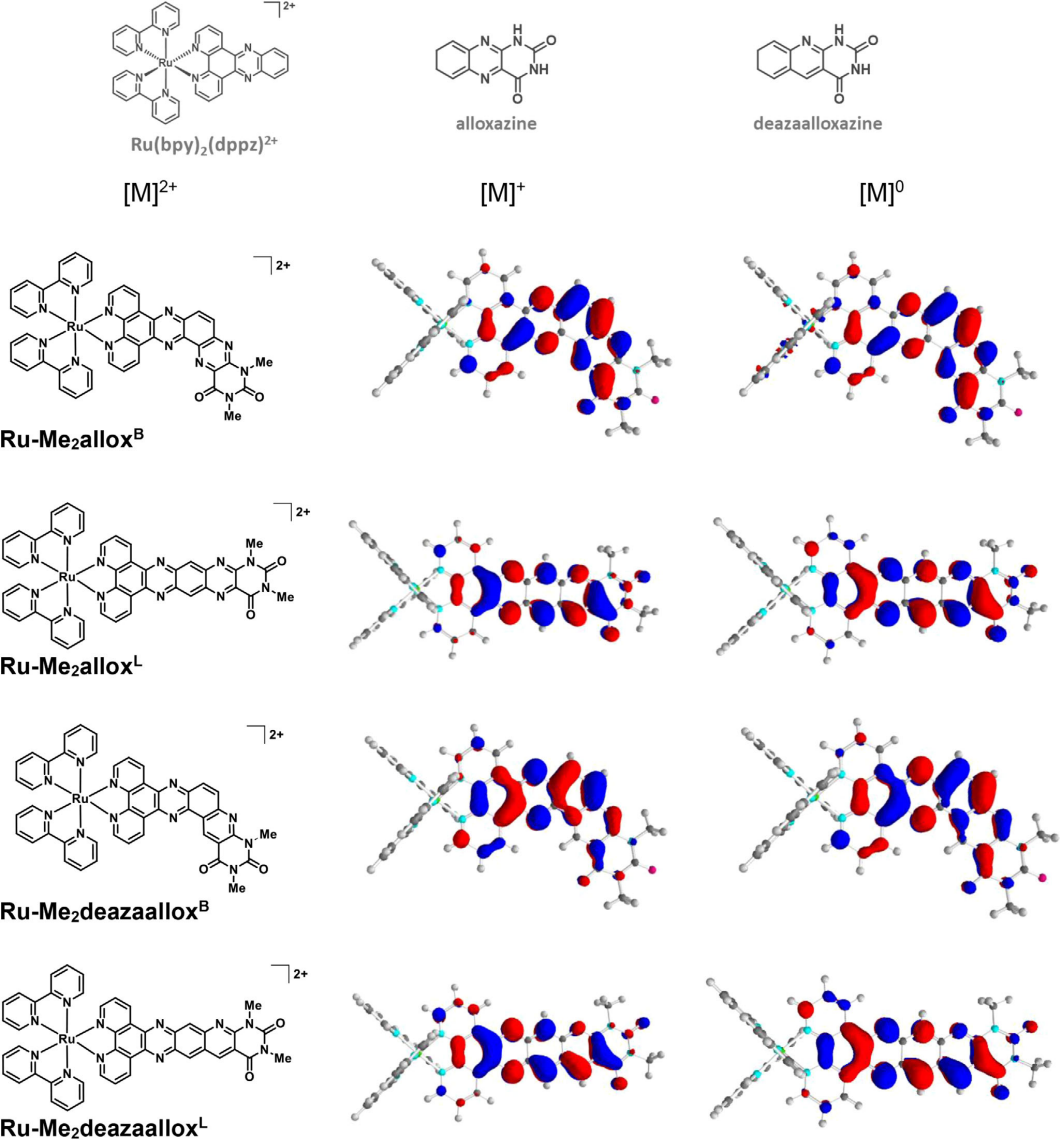
Structures of computationally investigated alloxazine and deazaalloxazine‐based complexes (left), together with the frontier orbitals (density value of 0.03 a.u.) for the (spin α) singly occupied molecular orbital of the singly reduced derivatives ([M]^+^, middle) and the highest occupied molecular orbital of the doubly reduced derivatives in their singlet state ([M]^0^, right; bonding energies for the singlet and triplet states given in Table S2). Structures of the [Ru(bpy)_2_(dppz)]^2+^ scaffold and the alloxazine/deazaalloxazine subunits are shown in the upper section.

Four Ru complexes featuring a flavin‐inspired π‐extended ligand (Figure 1) were computationally investigated by DFT and TDDFT simulations with respect to their electrochemical and photophysical properties prior to synthesis. They all share the [Ru(bpy)_2_(dppz)]^2+^ scaffold, fused either linearly or in a bent mode with a (deaza)alloxazine subunit. The latter was selected because it has been reported that alloxazines are reduced at more negative potentials than their naturally occurring isoalloxazine counterparts,^[^ ^3^, ^6^ ^]^ which may be advantageous for future applications. In the given structures, the two amino groups on the terminal uracil ring are methylated in order to prevent photoinduced tautomerization of (deaza)alloxazine to (deaza)isoalloxazine, reported to occur via an excited‐state proton transfer mechanism;^[^ ^23^, ^24^, ^25^ ^]^ if this process takes place, it would strongly impact the spectroscopic and photophysical characterization.

The relative first and second reduction potentials for the four proposed complexes were calculated (see computational details in the Supporting Information, and Tables S1,S2) following the same approach as in our previous study,^[^ ^21^ ^]^ in order to predict how the nature of the π‐extended ligand (allox vs. deazaallox) and its geometry (bent vs. linear) would affect the redox properties along the series of the complexes. The calculated reduction potentials are given in Table 1 and the frontier orbitals corresponding to the singly occupied molecular orbitals (SOMO) for the singly reduced species [M]^+^ and to the highest occupied molecular orbitals (HOMO) for the doubly, reduced species [M]^0^ are drawn in Figure 1 (frontier orbitals corresponding to the lowest unoccupied molecular orbitals (LUMO) of [M]^2+^ shown in Figure S1 for comparison purpose). These results provide two important pieces of information:

1. There is a significant difference in the first reduction potential when comparing the linear structures to their bent analogues, the latter being predicted to be reduced at a potential 400 to 500 mV more negative (−1.17 V for **Ru‐Me_2_allox^B^** compared to −0.65 V for **Ru‐Me_2_allox^L^**). Similarly, the calculated second reduction potential is shifted cathodically (250 to 400 mV) for the bent structures. This observation is in perfect agreement with the experimental study by the group of F. McDonnell^[^ ^26^ ^]^ on dinuclear Ru complexes with poly‐*N*‐heterocyclic bridging ligands, in which the first and second reduction processes on the bent bridging ligand are reported to occur at potentials 500 mV and 430 mV more negative than those on the linear bridging ligand, respectively. The presence of the two nitrogen lone pairs pointing toward each other in the bent structure generates some repulsion, as can be also observed here for **Ru‐Me_2_allox^B^** (Figure 1); this has been put forward to explain this difference in energy, such a repulsion being certainly exarcerbated upon reduction of the ligand. In **Ru‐Me_2_deazaallox^B^**, a similar effect can originate from the hydrogen atom pointing toward the lone pair of the nitrogen. The bent geometries thus generate electronic repulsion and, consequently, the HOMO levels of the bent structures are calculated to be higher in energy than those of their linear counterparts by 0.4–0.5 eV.
2. On the other hand, there is no significant effect of the nature of the extended part of the ligand, *i.e*., alloxazine versus deazaalloxazine, with **Ru‐Me_2_allox^B^** and **Ru‐Me_2_deazaallox^B^** presenting very close calculated first and second reduction potentials (−1.17 and − 1.25 V vs. Fc^+^/Fc for the first reduction, −1.62 and − 1.67 V versus *F*c^+^/*F*c for the second reduction, respectively).

**Table 1:** DFT‐computed 1^st^ and 2^nd^ reduction potentials versus SHE and ferrocene (SHE values shifted by −0.64 V compared to *F*c values) in DMF for the four complexes together with the experimentally determined values (in V vs. *F*c^+^/*F*c) for **Ru‐Me_2_allox^B^** and **Ru‐Me_2_deazaallox^B^** (Supporting electrolyte: TBAPF_6_, 0.1 M in DMF).

Complex	Reduction	E°[eV]_SHE_	E°[eV]_Fc+/Fc_	Exp [V vs *F*c^+^/*F*c]
**Ru‐Me_2_allox^B^**	1^st^	−0.53	−1.17	−1.26
	2^nd^	−0.98	−1.62	−1.60
**Ru‐Me_2_allox^L^**	1^st^	−0.01	−0.65	―
	2^nd^	−0.57	−1.21	―
**Ru‐Me_2_deazaallox^B^**	1^st^	−0.61	−1.25	−1.33
	2^nd^	−1.03	−1.67	−1.80
**Ru‐Me_2_deazaallox^L^**	1^st^	−0.21	−0.85	―
	2^nd^	−0.78	−1.42	―

On this basis, **Ru‐Me_2_allox^B^** and **Ru‐Me_2_deazaallox^B^** are the two target complexes that have been selected to be synthesized.

### 2.2 Synthesis and Electrochemical Characterization of **Ru‐Me_2_allox^B^** and **Ru‐Me_2_deazaallox^B^**

**Ru‐Me_2_allox^B^** and **Ru‐Me_2_deazaallox^B^** were synthesized in four steps from 7‐amino‐dipyridophenazine (**7‐amino‐dppz**) with overall yields of 16% and 4%, respectively (Figure S2). In this precursor, the nucleophilic character of the amino group is significantly weakened by the extended dppz conjugation compared to aniline, a starting material classically employed in the synthesis of more simple (deaza)alloxazine structures; as a consequence, the nucleophilic substitution on either 6‐chloro‐3‐methyl‐5‐nitropyrimidine‐2,4(1H,3H)‐dione (alloxazine precursor) or 6‐chloro‐2,4‐dioxo‐1,2,3,4‐tetrahydropyrimidine‐5‐carbaldehyde (deazaalloxazine precursor) was not very effective, even at high temperature, nor was the subsequent cyclization yielding **Meallox^B^** (or **deazaallox^B^**). This has also prevented the development of a chemistry‐on‐the‐complex route,^[^ ^27^ ^]^ the amine nucleophilicity being even weaker in the [Ru(bpy)_2_(**7‐amino‐dppz**)]^2+^ complex (Figure S2). Complexation to the Ru center proved to be necessary to purify the mixture of **Meallox^B^** (or **deazaallox^B^**) and remove the noncyclized intermediate and unreacted **7‐amino‐dppz**; this was followed by the methylation of the NH groups on the terminal uracil ring.

Cyclic voltammetry was then used to investigate the redox properties of the two new complexes (Figure S3). On the anodic scan, the metal‐centered Ru^III/II^ process is observed at + 0.85 and + 0.83 V versus Fc^+^/Fc for **Ru‐Me_2_allox^B^** and **Ru‐Me_2_deazaallox^B^**, respectively. On the cathodic scan, the cyclic voltammogram (CV) of the alloxazine‐based complex **Ru‐Me_2_allox^B^** displays five successive reduction processes, at − 1.26, −1.60, −1.80, −2.02, and − 2.49 V versus Fc^+^/Fc, respectively. Of note, the redox potentials determined experimentally for the first two reductions closely match the calculated ones (Table 1), hence validating our approach. These two reductions are located on the alloxazine subunit of the π‐extended ligand, according to the calculated orbital isodensity surfaces (Figure 1), which is a first prerequisite for multiple charge accumulation on a single site. The three following processes are attributed to the successive one‐electron reductions of the diimine ligands, by comparison with the data we previously reported for the reference complexes [Ru(bpy)_3_]^2+^ and [Ru(bpy)_2_(dppz)]^2+^ as well as for a related system with a π‐extended ligand.^[^ ^19^ ^]^ In contrast, the CV of the deazaalloxazine counterpart **Ru‐Me_2_deazaallox^B^** only displays four successive reduction processes at − 1.33, −1.80, −2.02, and − 2.27 V versus *F*c^+^/*F*c, respectively (Figure S3). The first reduction is in good agreement with the calculated value; however, the second one shows a slightly larger discrepancy than the others (Table 1). According to the calculated orbital isodensity surfaces (Figure 1), the first reduction is more localized on the dppz part of the π‐extended ligand rather than on the deazaalloxazine subunit, which should make a second reduction on the same ligand more difficult. Taking a closer look at the computational data, it can be seen that, after the first reduction and within the *β* spin manifold, where the second reduction will occur, both, the LUMO and LUMO + 1 levels, are energetically close (< 0.1 eV). The LUMO orbital corresponds to a mixture of both bpy ligands (Figure S4, top/left) whereas the LUMO + 1 matches the π‐extended ligand (Figure S4, top/right). After the 2^nd^ reduction, the (now) HOMO level is located on the extended ligand (Figure 1) and the LUMO level appears as a mixture of bpy and extended ligands (Figure S4, bottom). Thus, there is a close energetic competition between the bpys and the π‐extended ligands for the 2^nd^ reduction and the outcome of the DFT computation (the second reduction occurs on the extended ligand rather than on bpy ligands) may depend on additional subtle factors not explored here. However, it should be noticed that the reduction potentials determined experimentally for **Ru‐Me_2_deazaallox^B^** are very close to the ones reported for the reference complex [Ru(bpy)_2_(dppz)]^2+^ (recorded in the same electrolyte, i.e., 0.1 M nBu_4_NPF_6_ in DMF), with a dppz‐centered reduction at − 1.36 V versus *F*c^+^/*F*c and a first bpy‐centered reduction at − 1.79 V versus *F*c^+^/*F*c.^[^ ^19^ ^]^ This observation tends to support that **Ru‐Me_2_deazaallox^B^** electrochemically behaves like [Ru(bpy)_2_(dppz)]^2+^ and that the benefit of the ligand extension with a flavin‐inspired subunit to store multiple charges is lost when switching from the allox series to the deazaallox one. The alloxazine‐based target complex thus appears to be the one having the most interesting electrochemical properties for future applications based on light‐driven multielectron storage on a single electron reservoir site. Hence, in the following, the detailed spectroscopic and photophysical characterization of **Ru‐Me_2_allox^B^** is described.

#### 2.2.1 Spectroscopic Characterization of **Ru‐Me_2_allox^B^**

**Figure 2:**
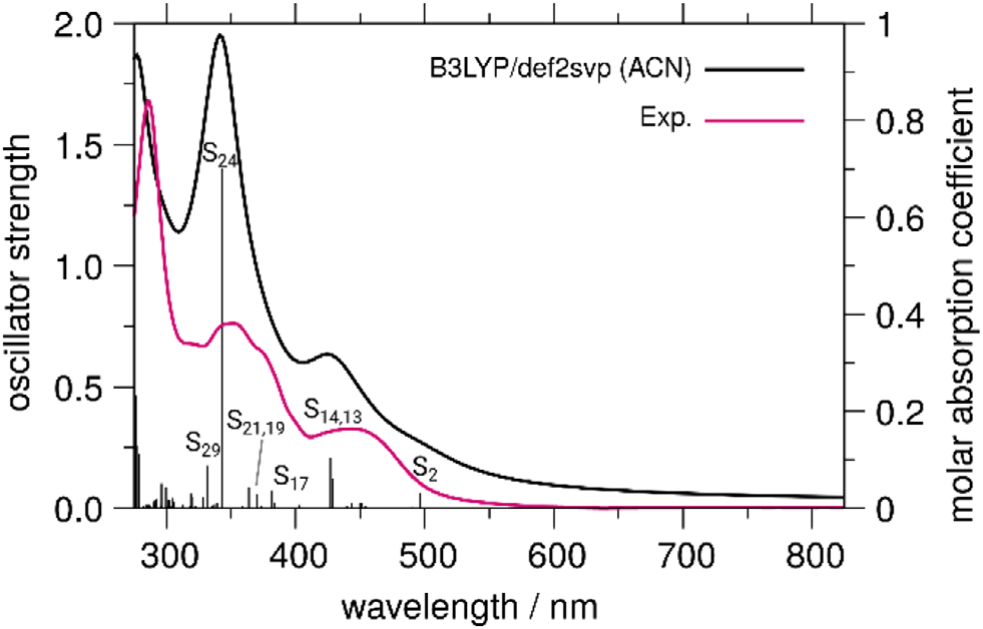
Comparison of experimental (pink) versus calculated (black) UV/Vis spectra for Ru‐Me_2_allox^B^ in its singlet ground state geometry. Electronic transitions (black vertical bars) represent underlying dipole‐allowed singlet–singlet excitations, broadened by Lorentzian functions with a full width at half maximum of 0.2 eV.

The absorption spectrum *of* ***Ru‐Me_2_allox^B^*** in acetonitrile (MeCN) (Figure 2) features a band at 285 nm caused by ligand‐centered transitions on the phenanthroline (phen)/bpy units^[^ ^28^, ^29^ ^]^ and a metal‐to‐ligand charge transfer (MLCT) band between 410–550 nm, which is characteristic for these type of Ru complexes. Additionally, **Ru‐Me_2_allox^B^** shows a structured band between 330–400 nm. To unravel the nature of the underlyingstates in both spectral regions DFT as well as TDDFT simulations were performed (Table S3 and S4). The band in the visible region of the spectrum, as already mentioned, stems from MLCT excitations toward the alloxazine ligand (into S_2_, 487 nm), the bpy coligands (into S_13_, 429 nm) as well as a combination of both (S_14_, 427 nm). Contributions to the spectral region between 330 and 400 nm originate from the alloxazine‐centered MLCT state S_19_ (370 nm) and alloxazine‐centered intraligand (IL) transitions, e.g., S_17_, S_21_, S_24,_ and S_29_ at 382, 364, 343, and 343 nm, respectively.

**Figure 3:**
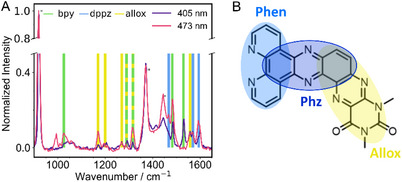
Resonance Raman spectra of Ru‐Me_2_allox^B^ upon excitation at 405 and 473 nm in MeCN (100–200 µM) normalized to the solvent peak at 920 cm^−1^ a). Solvent peaks are marked by an asterisk. Chemical structure of the extended ligand where the different structural moieties are highlighted b).

Resonance Raman spectroscopy was employed to obtain further insights into the nature of the observed optical transitions and to characterize the Franck‐Condon region after optical excitation at 405 and 473 nm. The corresponding experimental spectra are shown in Figure 3a.

Comparison to calculated spectra (Figure S5 and Table S5) and spectra of [Ru(bpy)_2_(dppz)]^2+^ (Figure S6) reveals that peaks associated with the bpy ligands (1027, 1317, 1480, and 1535 cm^−1^) and the phen and phenazine (phz) unit of the dppz moiety (1470, 1570, and 1590 cm^−1^) are found for both excitation wavelengths. The signals at 1170, 1195, 1270, and 1560 cm^−1^ are not observed in the [Ru(bpy)_2_(dppz)]^2+^ reference spectra (neither for 405 nor for 473 nm excitation) so that they are ascribed to the extension of the dppz‐like ligand. Hence, dipole‐allowed transitions related to an excitation involving the alloxazine are induced as well. With that, the data suggest that transitions comprising the entire ligand sphere are induced at both excitation wavelengths. Despite that, the spectra shown in Figure 3 demonstrate that the Franck‐Condon states populated upon excitation at 473 nm and 405 nm differ. The peak at 1535 cm^−1^ is, for example, only present upon excitation at 405 nm and the relative intensity of the peaks changes when shifting the excitation wavelength. The peak intensity at 1560 cm^−1^ (after normalization to the solvent peak at 920 cm^−1^) is not affected by the change in the excitation wavelength and is taken as point of reference. Peaks with increasing relative intensity can be ascribed to the bpy (1535 cm^−1^) as well as the dppz (1595 cm^−1^) and alloxazine (1170 cm^−1^) units. This points toward the fact that a mixture of optical transitions with different characters (e.g., MLCT_allox_, MLCT_bpy_…) contributes to each spectral region. This conclusion agrees with the above‐mentioned optical transitions predicted by the performed TDDFT simulations. In particular, the excited states S_2_, S_13_, and S_14_, which involve the extended ligand, the bpy ligands and the phen unit, are expected to be populated upon excitation at 473 nm. The two latter states are likely populated upon excitation at 405 nm, too, causing peaks associated with vibrational normal modes of the bpy and dppz moieties. While a transition to the MLCT_allox_ state S_2_ is found at longer wavelengths and rather not induced upon excitation at 405 nm, the alloxazine peaks observed at this wavelength are related to the population of mainly two IL transitions (S_17_ and S_21_) as well as one MLCT transition (S_19_).

#### 2.2.2 UV/Vis Spectroelectrochemistry and EPR Characterization of Singly Reduced **Ru‐Me_2_allox^B^**

**Figure 4:**
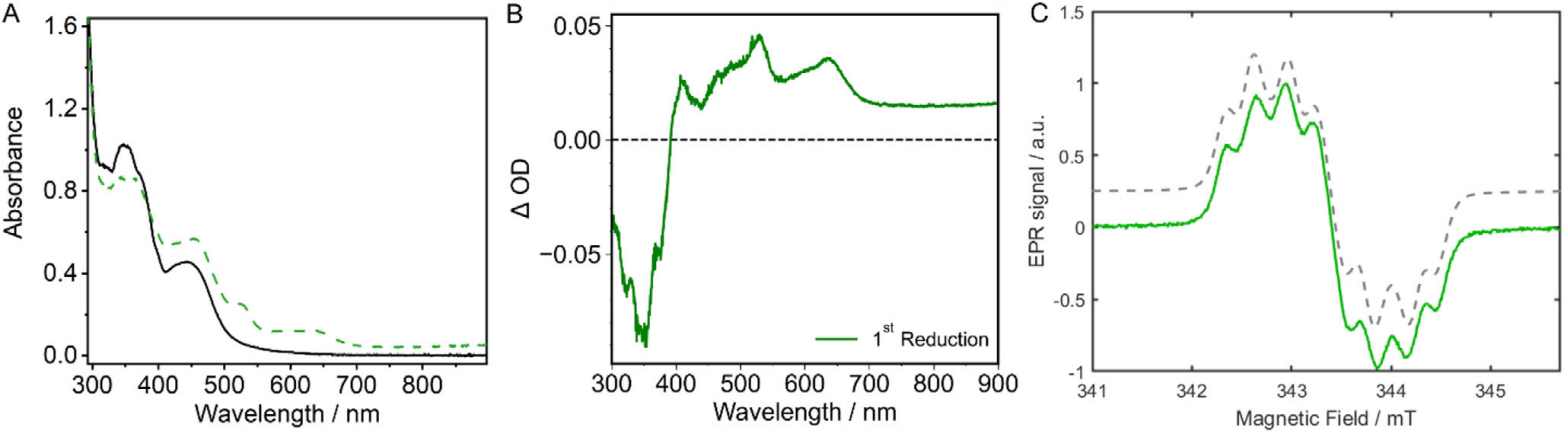
UV/Vis spectra of neutral (black line) and singly reduced (green dashed line) **Ru‐Me_2_allox^B^** in acetonitrile; one equivalent of cobaltocene was used as reductant a). UV/Vis difference absorption spectrum of **Ru‐Me_2_allox^B^** at an applied potential corresponding to the first reduction in acetonitrile; The electrolyte contained 0.1 M TBAPF_6_ (glassy carbon working electrode, Pt counter electrode, and Ag/AgCl pseudo‐reference electrode) b). EPR spectrum of singly reduced **Ru‐Me_2_allox^B^** (green line) and the corresponding simulation (grey dashed line); one equivalent of cobaltocene was used as reductant c).

In addition to the neutral complex, the singly reduced derivative of **Ru‐Me_2_allox^B^** was spectroscopically and theoretically investigated. This compound was prepared by chemical reduction with cobaltocene (see Supporting Information for experimental details) and the resulting UV/Vis absorption spectrum (Figure 4a) is in agreement with the difference spectra recorded by means of spectroelectrochemical (SEC) measurements (Figure 4b). More specifically, an increase of the MLCT absorption between 400–500 nm is observed and a shoulder around 525 nm as well as a broad band around 630 nm evolve. Of note, keeping a reactive MLCT state is an important feature in the aim of driving a second reduction process with light, as required for systems performing charge photoaccumulation.^[^ ^19^, ^26^ ^]^

Quantum chemical calculations, again, allowed us to assign discrete states to these spectral features. The newly formed band at 620–640 nm is attributed to the mixed ILCT and LLCT state D_10_ (574 nm), which shifts electron density within the π‐extended alloxazine ligand and toward the bpy coligands of the metal center (Figure S7, Tables S6 and S7). The band at 510–525 nm in the experimental spectrum is assigned to the D_18_ state (494 nm), which represents an ILCT from the alloxazine subunit toward the phz moiety closer to the metal center. The electronic structure of the singly reduced derivative was further investigated by X‐band EPR spectroscopy (see Supporting Information for experimental details).

The singly reduced species exhibits a very intense EPR signal, with a *g*‐value characteristic of an organic radical (*g* = 2.003) (Figure 4c, black line). Upon close inspection, the EPR signal exhibits several hyperfine coupling splittings. This spectrum can be successfully simulated by a paramagnetic species with four hyperfine couplings originating from two nitrogen atoms (spin 1 nucleus, hyperfine coupling constants of 11.2 MHz and 9.4 MHz) and 2 protons (spin ½ nucleus, hyperfine coupling constants of 6.8 MHz and 5.7 MHz) (Figure 4c, grey dashed line), in excellent agreement with the calculated isodensity surface for the SOMO orbital of singly reduced **Ru‐Me_2_allox^B^** (see Figure 1). Moreover, the experimentally determined hyperfine values have been successfully reproduced by DFT with two nitrogen hyperfine couplings at 11.0 and 8.4 MHz and two proton hyperfine couplings at 7.1 MHz and 6.9 MHz (Table S8).

#### 2.2.3 Photoinduced Processes in **Ru‐Me_2_allox^B^**

**Figure 5:**
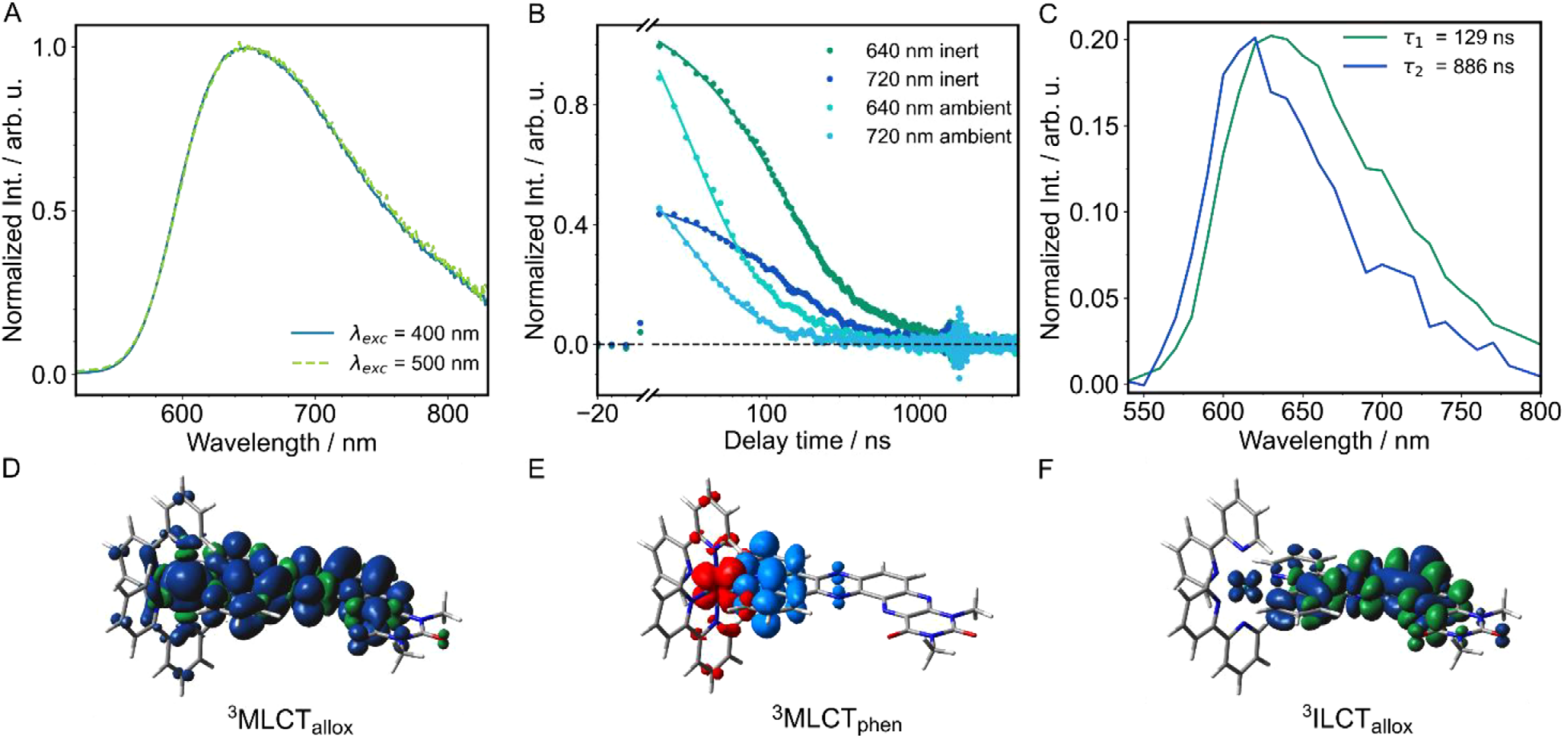
Emission spectrum of Ru‐Me_2_allox^B^ in MeCN upon excitation at 400 nm and 500 nm a). Kinetic traces of the time‐resolved emission of Ru‐Me_2_allox^B^ in MeCN at 640 and 720 nm in the absence and presence of oxygen upon excitation at 450 nm b) and corresponding spectra of each species obtained from a biexponential global fit c). The latter are given for the measurements under inert conditions but are representative for the measurements under ambient environment as well. Spin densities for the three low‐lying states MLCT_allox_ d), MLCT_phen_ e), charge density difference plot, and ILCT_allox_ f), which were determined using TDDFT. All xyz‐structures are available via the online repository Zenodo.^[^ ^34^ ^].^

The complex exhibits emission above 550 nm (Figure 5a). The emission quantum yield was measured using an integrating sphere but was found to be below a reliably measurable value with the given system (below 0.5 %). The emission spectrum is featureless and the emission maximum lies at 650 nm. It is known that Ru polypyridyl complexes undergo very fast ISC (< 100 fs) and the emission of the parent complexes [Ru(bpy)_3_]^2+^ and [Ru(bpy)_2_(dppz)]^2+^ is found around 620 nm. Based on these considerations in combination with results on a related Ru complex recently reported by us,^[^ ^11^ ^]^ we expect the emission to stem from radiative decay of a triplet state. To support this hypothesis, the lowest‐lying triplet states of **Ru‐Me_2_allox*^B^*** have been investigated by means of TDDFT (with singlet‐triplet transitions). Notably, scalar‐relativistic TDDFT (including spin‐orbit‐couplings) simulations, as performed within the Franck‐Condon geometry, suggest such fast ISC between the singlet and triplet MLCT states, while triplet states on the π‐extended ligand, i.e., the ^3^ILCT_allox_ state (T_2_), does not contribute significantly to the singlet‐triplet population transfer. This is in agreement with similar 4d and 5d transition metal complexes^[^ ^30^, ^31^, ^32^, ^33^ ^]^ with π‐extended ligands (see Table S9 for spin‐orbit couplings). The emissive T_1_ state of **Ru‐Me_2_allox*^B^*** is found to be of ^3^MLCT nature with excitation toward the alloxazine moiety (^3^MLCT_allox_, Figure 5d). The π‐extended alloxazine ligand shares this behavior with the smaller dppz‐like alloxazine ligand recently investigated by us, where the emissive T_1_ state was found to be of MLCT nature with excitation toward the alloxazine moiety, too.^[^ ^11^ ^]^ The predicted emission wavelength of 630 nm (1.97 eV, see Table S10) for the transition from the T_1_ state to the singlet ground state matches the experimental data.

Time‐resolved emission measurements in the sub‐µs range were conducted to obtain further information about the bright excited‐state(s) (Figure 5 and Figure S8). As these states are often long‐lived in Ru complexes, they are expected to play a crucial role in the photoreaction leading to charge accumulation. Analysis of the kinetic traces (Figure 5b) reveals that two relaxation pathways to the ground state exist as the decay of the signal is biexponential. Under ambient atmosphere, a global fit of the data using a sum of two exponentials yields characteristic time constants *τ*_1,EmAir_ = 30 ns and *τ*_2,EmAir_ = 194 ns. The corresponding emission maxima are found to be 630 and 615 nm, respectively, as evident from the decay‐associated spectra (DAS) (Figure S8C). The measurements were additionally performed in the absence of oxygen to verify our above hypothesis that the emission originates from triplet states. Such triplet states should be quenched by triplet oxygen resulting in a shorter lifetime compared to inert conditions. Indeed, longer time constants of *τ*_1,Em_ = 129 ns and *τ*_2,Em_ = 886 ns (with similar DAS as before) are obtained for the measurements in the degassed solvent (Figure 5b,c).

Hence, we conclude that two triplet states are populated and decay back to the ground state on a sub‐µs timescale. Assuming that the quenching follows a Stern‐Volmer behavior, equation 1 can be applied. With that, the lifetimes given above demonstrate that the quenching rate for the emissive state linked to *τ*_1_ is higher than for the one linked to *τ*_2_.

(1) $$\frac{1}{\tau }-\;\frac{1}{\tau_{0}}=k_{q}\left[Q\right]$$

Quantum chemical calculations allow us to identify three potential low‐lying triplet states that may rationalize the experimental findings. The aforementioned T_1_ state of ^3^MLCT_allox_ character was obtained by optimization via DFT. As stated above, its emission wavelength of 630 nm is in perfect agreement with the maximum of the emissive state linked to *τ*_1_ around 630 nm. The fact that it is redshifted compared to [Ru(bpy)_2_(dppz)]^2+^ and [Ru(bpy)_3_]^2+^ gives experimental evidence that the extended part of the ligand is involved. The ^3^MLCT_allox_ character of this state is further supported by fs‐TA measurements, which were carried out to get more insights into the formation of the long‐lived excited states and which will be discussed in the next section. Optimizing the T_2_ state (^3^ILCT_allox_, Franck‐Condon point; Figure 5f) by the means of excited‐state tracking using TDDFT and our external optimizer pysisyphus^[^ ^35^ ^]^ we found that this T_2_ relaxes to the lowest energy triplet state within its equilibrium structure. The state shows π‐π* character within the entire alloxazine ligand with a predicted emission wavelength of 682 nm (Table S10). The redshifted emission compared to ^3^MLCT_allox_ makes it an unlikely candidate for the second emissive state which exhibits an emission maximum of ∼ 615 nm.

In contrast, by tracking the third triplet excited state with TDDFT, we found another likely candidate for the emissive state, which was only accessible via the optimized ^3^MLCT_phen_ state. The still state — also of ^3^MLCT nature (T_3_) — features excitation predominantly toward the phen‐moiety (Figure 5e) of the extended ligand. The emission wavelength was predicted to be 611 nm, which is much closer to the observed emission maximum (Table S10). Moreover, the DAS matches the steady‐state emission spectrum of [Ru(bpy)_3_]^2+^ and the emission maximum as well as the lifetime represented by *τ*_2_ is very similar to what has been reported^[^ ^36^, ^37^, ^38^ ^]^ for [Ru(bpy)_2_(dppz)]^2+^ in which emission stems from a phen‐centered state.^[^ ^39^ ^]^ Based on these considerations and the calculations, we assign the emission linked to *τ*_2_ to a similar state (^3^MLCT_phen_; Figure 5e) where the charge is more localized on the proximal part of the extended ligand.

To conclude, the observed emission is attributed to two emissive ^3^MLCT excited states — one involving the phen sphere and the other one associated with the alloxazine ligand sphere. Notably, the ^3^MLCT_phen_ does not relax to the lowest excited state in its equilibrium structure. Therefore, the emission from the ^3^MLCT_phen_ state is likely related to a non‐Kasha MLCT emission. An explanation why the longer excited‐state lifetime (*τ*_2_) is linked to the higher‐lying excited state (^3^MLCT_phen_) could lie in the involvement of different d‐orbitals in the ^3^MLCT_phen_ and the ^3^MLCT_allox_ state (Figures 5e and 7, Tables S11 and S12). The required change of the electron density on the metal center might prevent efficient conversion from the phen‐centered to the alloxazine‐centered state.

Femtosecond TA spectroscopy was performed to characterize the excited‐state dynamics of **Ru‐Me_2_allox*^B^*** and, as mentioned above, to learn more about the formation of the long‐lived excited states to deduce possible implications for the complex's light‐driven reactivity.

**Figure 6:**
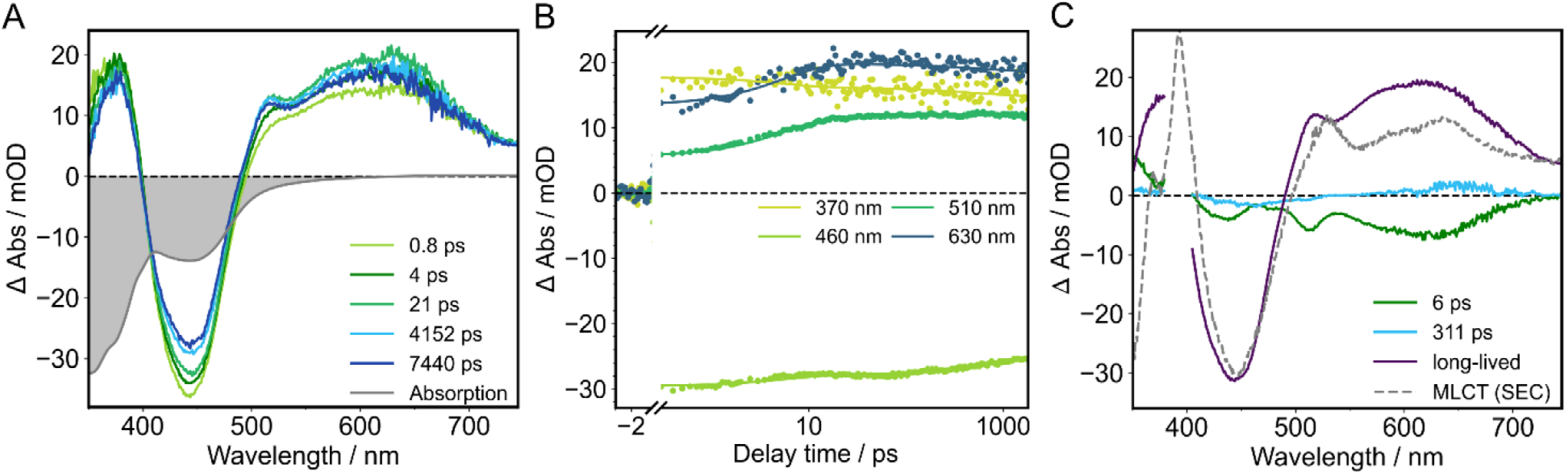
Transient absorption spectra of **Ru‐Me_2_allox^B^** in MeCN measured at different delay times after excitation with a 400‐nm pulse a), kinetic traces with corresponding fit at selected wavelengths b) and decay‐associated spectra (DAS) obtained by a global fit of the data c). The dashed, grey line represents the approximation of the MLCT state via spectroelectrochemistry.

The following part outlines the excited‐state processes of **Ru‐Me_2_allox*^B^*** in MeCN and butyronitrile (BuCN) upon optical excitation at 400 nm on a sub‐nanosecond timescale. Considering the absorption and rR data, a mixture of IL and MLCT transitions is expected at this excitation wavelength. The measurements were performed under ambient environment, i.e., in nondegassed solvents. The overall shape of the spectra (Figure 6 and Figure S9) is dominated by an excited‐state absorption (ESA) below 390 nm, a ground‐state bleach (GSB) between 400 and 490 nm and a broad absorption band above 500 nm. The latter comprises a prominent shoulder at 520 nm, which evolves on a picosecond timescale, as well as an ESA maximum around 630 nm. As expected from the time‐resolved emission measurements, the compound shows a lifetime which is longer than the 8 ns range accessible by the setup.

For a quantitative analysis, a multiexponential global fit was applied to the data sets. The measured spectra are best fitted by a sum of three exponentials. Due to the ultrafast intersystem crossing of Ru polypyridyl complexes, ^[^ ^40^ ^]^ which cannot be covered with the time resolution of our setup, we expect all observed states to be of triplet character.

UV/Vis SEC absorption measurements of the reduced species, discussed above (Figure 4), and the oxidized species (Figure S10) are used to assign spectral features to the lowest excited MLCT state.^[^ ^41^, ^42^ ^]^ According to literature^[^ ^19^ ^]^ and the DFT calculations outlined above the oxidation is metal‐centered (Ru^II^/Ru^III^) whereas the first reduction involves an orbital on the extended ligand. This translates to the HOMO of **Ru‐Me_2_allox*^B^*** being centered on the metal center and the LUMO being associated with the extended ligand. The lowest‐lying MLCT transition involves the same orbitals, as confirmed by the calculations, and it has been shown that its spectral features can often be simulated by a linear combination of the spectrum of the singly reduced and oxidized species.^[^ ^41^ ^]^

Upon oxidation, **Ru‐Me_2_allox*^B^*** shows a bleach of the MLCT band between 400 and 550 nm and the evolution of a double peak in the region 360–400 nm is observed. The decrease of the MLCT band originates from the fact that the electron density at the Ru center is reduced upon oxidation, which prevents the charge shift from the metal to the ligand found in the neutral species. The most striking spectral features of the singly reduced species are a new band at around 620–640 nm and a shoulder at 510–525 nm (vide supra).

**Figure 7:**
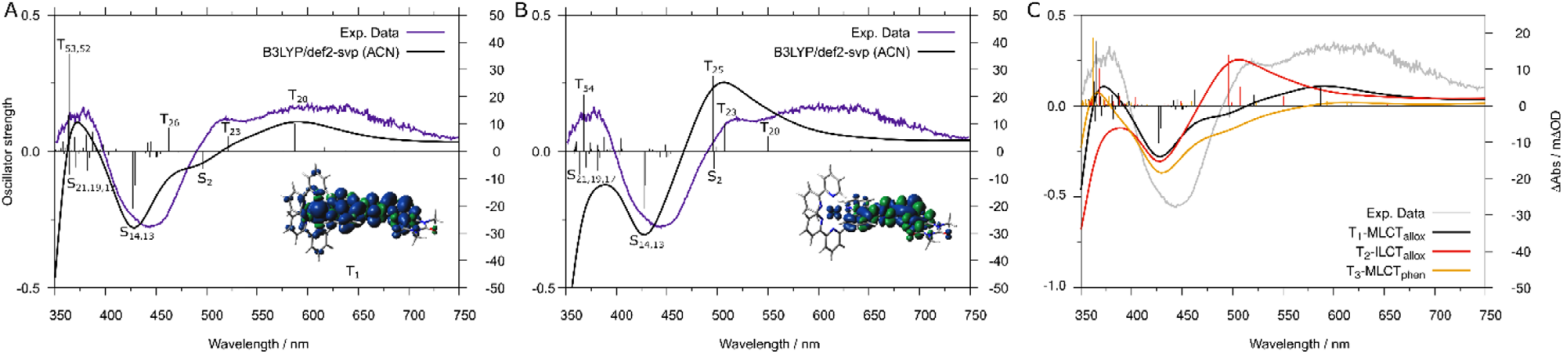
Calculated transient absorption (TA) spectrum for **^3^Ru‐Me_2_allox^B^** a). Excitation occurs from a charge‐separated ^3^MLCT_allox_ state with electron density on the alloxazine moiety. The spin density for the described ^3^MLCT_allox_ state is given in the image as T_1_. Electronic transitions (black vertical bars) represent underlying dipole‐allowed singlet‐singlet (negative) and triplet‐triplet (positive) excitations, broadened by Lorentzian functions with a full width at half maximum of 0.2 eV. Calculated TA spectrum for **^3^Ru‐Me_2_allox^B^** b). Excitation occurs from a ^3^IL state on the alloxazine moiety. Comparison of different calculated TA spectra c). Excitation occurs respectively from the optimized T_1_ (black trace, ^3^MLCT_allox_), T_2_ (red trace, ^3^ILCT_allox_), or from the underlying T_2_ state under the optimized T_3_ state (yellow trace, ^3^MLCT_phen_).

The TA spectrum of the long‐lived species is characterized by a GSB around 450 nm and an ESA with a maximum at 620 nm and a shoulder at 520 nm in both solvents. Following the described approach for the approximation of MLCT spectral features via spectroelectrochemistry, the prominent spectral features of the long‐lived species of **Ru‐Me_2_allox*^B^*** were recreated by the linear combination of the difference absorption spectra of the electrochemically oxidized and reduced complex (Figure 6c). The results are in agreement with the time‐resolved emission measurements and theoretical predictions by unrestricted DFT regarding the lowest excited state (vide supra) and confirms the assignment of one of the long‐lived emissive MLCT states to ^3^MLCT_allox_. Additional insight into the nature of the spectral features of the triplet excited state could be gained by employing TDDFT (Figure 7, Tables S11 and S12). The vertical excitations from the lowest accessible triplet excited state T_1_ (^3^MLCT_allox_) were calculated and the TA spectrum obtained in this way is in good agreement with the spectra measured during the time‐resolved absorption experiments (Figure 7a). The ESA band above 500 nm in the experiment is mainly caused by a transition to the T_20_ state (587 nm) (Figure 7a), which features IL charge redistribution within the alloxazine ligand. Two additional alloxazine centered ILCT states contribute to the spectral shape in this region — the T_23_ (521 nm) and the T_26_ (463 nm) triplet excited states, the first of which gives rise to the shoulder at 520 nm in the experimental TA spectrum, whereas the second state is concealed by the GSB between 400 and 500 nm. The fact that the spectrum approximated by SEC measurements (Figure 6c) does not perfectly reproduce the intensity ratio between the shoulder at 520 nm and the broad band around 620 nm points toward a mixture of species contributing to the TA spectra at long delay times. This is in line with the biexponential decay of the emission signal discussed above.

**Figure 8:**
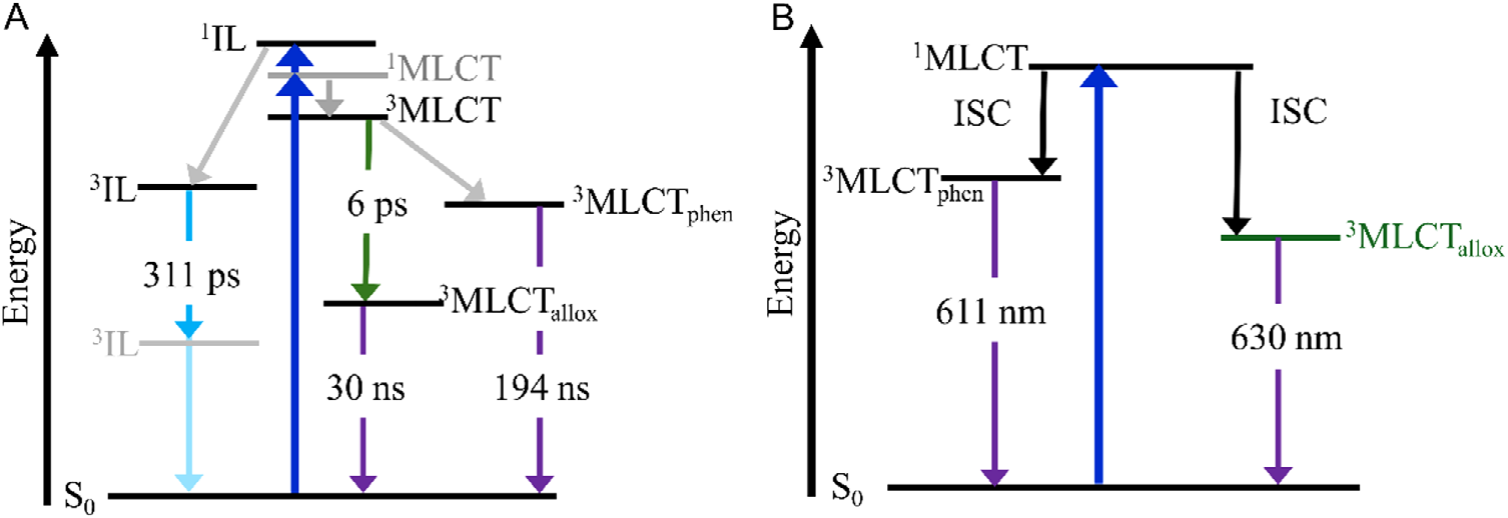
Jablonski diagram for the suggested relaxation pathway of Ru‐Me_2_allox after excitation at 400 nm (deduced from the experimental data) a). Possible relaxation pathways in the MLCT manifold which could lead to radiative decay, as predicted by excited‐state tracking using TDDFT b).

Apart from long‐lived excited states, the global analysis infers the existence of a fast process with a first‐order time constant *τ*_1_ = 6 ps. The corresponding DAS mirrors the features found above 500 nm in the DAS/TA spectra of the long‐lived species, i.e., a shoulder around 520 nm and a broad band with a maximum around 630 nm. Therefore, the first process is assigned to formation of the ^3^MLCT_allox_ state (Figure 8). The rR measurements confirmed that charge is transferred to both, the bpy as well as the extended ligand, at the Franck‐Condon point. Consequently, *τ*_1_ describes a charge shift from the entire ligand sphere to the extended alloxazine‐based ligand. Additionally, the fact that the process is not accompanied by an enhancement of the bleach around 450 nm corroborates that the process takes place in the MLCT manifold, i.e., the ^3^MLCT_allox_ state is populated from another ^3^MLCT state where the Ru center is already oxidized. The assignment of *τ*_1_ is supported by TA measurements in BuCN (*ε* = 20.7) which yield a longer *τ*_1_ of 12 ps (Figure S9) compared to MeCN (*ε* = 37.5). In the latter solvent, the ^3^MLCT_allox_ state is expected to be more stabilized than the other states due to the longer distance between the positive and negative charge in the charge‐separated state, which renders it more polar. This results in a higher driving force for the charge shift.

The multiexponential fit of the data in MeCN further yields a characteristic time constant of 311 ps. The DAS associated to *τ*_2_ has a low amplitude and indicates decreasing ESA above 550 nm and increasing absorption in the region 410–520 nm. According to TDDFT, one of the lowest‐lying excited states is of ^3^IL character (Figure 7b,c) so that *τ*_2_ is potentially linked to the ^3^IL manifold and describes relaxation via IL states in addition to the MLCT manifold.

In BuCN, a value of 272 ps is obtained for *τ*_2_ meaning that the effect of solvent polarity on this process is rather small compared to the effect that was observed for *τ*_1_. A polar MLCT state would be expected to show polarity‐dependent behavior whereas the rather unpolar nature of the ^3^IL is in line with the observation. In addition, ligand‐centered triplet states on π‐extended ligands oftentimes display weak GSB between 400–470 nm which is reflected in the increase in signal intensity in that region illustrated by DAS2 (Figure 6c and Figure S9C).

Despite these aspects which point toward observation of an IL state process, assignment of *τ*_2_ to the ^3^MLCT manifold cannot be fully ruled out. It is equally feasible to ascribe the characteristic time constant to relaxation of the ^3^MLCT_phen_ state, which theory predicts to be involved in one of the relaxation pathways and which was discussed as one of the long‐lived states in the framework of the time‐resolved emission measurements (Figure 8b).

## 3 Conclusion

This joint experimental and theoretical approach successfully enabled the design and investigation of photoactive ruthenium complexes featuring a flavin‐inspired redox‐active ligand. The synthesis of two new complexes was guided by DFT calculations of the redox potentials as a relevant variable for predicting electron properties of a series of target complexes. Especially for **Ru‐Me_2_allox^B^**, the first two reductions are found at more negative reduction potentials than for our previous complexes^[^ ^19^, ^21^ ^]^ and the calculated values closely match the reduction potentials determined from cyclic voltammetry (−1.17 V vs. −1.26 V for the 1^st^ reduction and −1.62 V vs. −1.60 V for the 2^nd^ reduction). Characterization of its singly reduced derivative via EPR and UV/Vis absorption spectroscopy and spectroelectrochemistry as well as (TD)DFT showed that the additional charge is localized on the extended ligand confirming the vital role of the alloxazine motif for the electrochemistry of the complex. The photophysical characterization of **Ru‐Me_2_allox^B^** revealed long lifetimes of the excited state extending to several hundreds of nanoseconds. Two ^3^MLCT states and possibly a ^3^IL state contribute to the excited‐state dynamics at long delay times after excitation. In contrast to our previously reported Ru(II) complex featuring an alloxazine unit, the extension of the ligand in **Ru‐Me_2_allox^B^** leads to a longer excited‐state lifetime and a more promising electrochemistry where both of the first two reductions are localized on the extended ligand. Based on the electronic properties described herein, we expect **Ru‐Me_2_allox^B^** to be capable of storing photogenerated charges at more reducing potentials than the previous generations of Ru complexes we reported and the investigation of its light‐driven reactivity is currently underway. If successful, the newly design photosensitizer, inspired from the natural flavin cofactors, is a very promising candidate for the light‐driven production of solar fuels.

## Conflict of Interests

The authors declare no conflict of interest.

## Supporting information



Supporting Information

## Data Availability

All optimized equilibrium structures are openly available in Zenodo at https://doi.org/10.5281/zenodo.15303442, reference number. ^[^ ^34^ ^]^
